# Technological Advancement in Tower-Based Canopy Reflectance Monitoring: The AMSPEC-III System

**DOI:** 10.3390/s151229906

**Published:** 2015-12-19

**Authors:** Riccardo Tortini, Thomas Hilker, Nicholas C. Coops, Zoran Nesic

**Affiliations:** 1 Integrated Remote Sensing Studio, Department of Forest Resources Management, University of British Columbia, 2424 Main Mall, Vancouver, BC V6T 1Z4, Canada; nicholas.coops@ubc.ca; 2 College of Forestry, Oregon State University, 231 Peavy Hall, Corvallis, OR 97333, USA; thomas.hilker@oregonstate.edu; 3 Faculty of Land and Food Systems, Univeristy of British Columbia, 2357 Main Mall, Vancouver, BC V6T 1Z4, Canada; zoran.nesic@ubc.ca

**Keywords:** gross primary production, photosynthesis, light use efficiency, remote sensing, PRI, multi-angle spectroscopy, spectro-radiometer

## Abstract

Understanding plant photosynthesis, or Gross Primary Production (GPP), is a crucial aspect of quantifying the terrestrial carbon cycle. Remote sensing approaches, in particular multi-angular spectroscopy, have proven successful for studying relationships between canopy-reflectance and plant-physiology processes, thus providing a mechanism to scale up. However, many different instrumentation designs exist and few cross-comparisons have been undertaken. This paper discusses the design evolution of the Automated Multiangular SPectro-radiometer for Estimation of Canopy reflectance (AMSPEC) series of instruments. Specifically, we assess the performance of the PP-Systems Unispec-DC and Ocean Optics JAZ-COMBO spectro-radiometers installed on an updated, tower-based AMSPEC-III system. We demonstrate the interoperability of these spectro-radiometers, and the results obtained suggest that JAZ-COMBO can successfully be used to substitute more expensive measurement units for detecting and investigating photosynthesis and canopy spectra. We demonstrate close correlations between JAZ-COMBO and Unispec-DC measured canopy radiance (0.75 ≤ R^2^ ≤ 0.85) and solar irradiance (0.95 ≤ R^2^ ≤ 0.96) over a three month time span. We also demonstrate close agreement between the bi-directional distribution functions obtained from each instrument. We conclude that cost effective alternatives may allow a network of AMSPEC-III systems to simultaneously monitor various vegetation types in different ecosystems. This will allow to scale and improve our understanding of the interactions between vegetation physiology and spectral characteristics, calibrate broad-scale observations to stand-level measurements, and ultimately lead to improved understanding of changing vegetation spectral features from satellite.

## 1. Introduction

Measurement and quantification of photosynthesis and other plant physiological processes are crucial to improve our understanding of ecosystem functioning. Tower-based approaches, such as Eddy-Covariance (EC) measurements, are critical to determine the exchange of carbon dioxide (CO_2_) between land surface and atmosphere, and to improve our understanding of cycling of water, nutrients, and carbon [1]. However, EC can only provide spatially discrete observations typically over a few hundred meter or few kilometer footprint, and upscaling these observations to larger areas is difficult. As a result, the prediction of carbon, water, and energy balances at landscape, regional and global scale is highly uncertain [2,3,4,5].

As a complementary technique to EC measurements, near surface remote sensing is an effective tool to link carbon fluxes and spectral features of vegetation across various spatial scales, from proximal [6,7,8,9] to airborne [10] and satellite platforms [11,12]. High-spectral and spatial resolution optical sensors can be utilized to determine physiological processes through the relationship between plant physiological properties and biochemical composition of foliage (e.g., [13]), typically observed through narrow spectral channels, in the 400–2500 nm range [14].

At the same time, optical remote sensing is also capable of synoptic coverage of the globe via satellite observation, hence providing opportunities for spatially continuous scaling of ecosystem fluxes (e.g., [15,16,17,18,19,20]). Network approaches, such as the SpecNet initiative [21], have demonstrated their effectiveness in linking remote measurements to carbon fluxes. Nonetheless, determination of highly dynamic and spatially variable physiological processes remains challenging, as a number of external factors can affect the relationship between biophysical properties and measured reflectance, including sun-view geometry, soil background reflectance, species and canopy characteristics and pigment pool size (e.g., [22,23,24,25,26,27]). Spatial and spectral dynamics of some of these processes requires high spatial and temporal resolution [28], and the observation of vegetation status under multiple illumination and viewing conditions (e.g., [29,30]), which is not easily achieved with existing airborne or spaceborne sensors [31]. Indeed, in order to detect pigment absorption features both high spectral resolution (in the range of few nm) and high signal-to-noise is needed [7].

Networking multi-angular tower observations based on field spectro-radiometers can help to provide a better understanding of ecosystem dynamics [7,8]. In particular, low cost spectro-radiometers developed over recent years may help overcome the often significant cost in purchasing these instruments and, thus, limiting the implementation of networks, which would permit more systematic upscaling of tower measured reflectance and fluxes to space. However, differences in data quality between these “off the shelf” radiometers compared to well established models is not clear nor is the impact on physiological indices derived from the data. In this paper we address this issue by undertaking a comparison between two portable spectro-radiometers, both installed on a third generation Automated Multiangular SPectro-radiometer for Estimation of Canopy reflectance system (AMSPEC-III). We discuss the system’s hardware and software components, as well as the results of the comparison of measured spectra from a three-month field test.

## 2. Materials and Methods

### 2.1. The AMSPEC-III System

The upgrades implemented in AMSPEC-III were based on previous experience over the past 5 years (*cf*. [7,8]). In order to reduce communication issues in the previous design, the AMSPEC-III consists of a single module mounted atop of the tower and controlled by a relay switch to allow remote power cycling of the system.

The tower module enables the direct comparison of two different portable spectro-radiometers: a Unispec-DC (UDC; PP-Systems, Amesbury, MA, USA) and a JAZ-COMBO (JC), formed by two JAZ-S and one JAZ-DPU (Ocean Optics, Dunedin, FL, USA), which technical specifications are described in Table 1. The spectro-radiometers were coupled through optical fibers, with an upward-looking sensor featuring a cosine diffuser (PP-Systems) to correct sky irradiance for varying solar altitudes. A webcam (NetCam SC 5MP, StarDot, Buena Park, CA, USA) image is automatically acquired simultaneously to spectra being co-registered in order to allow phenological assessment [32] and canopy shading from digital photography. The system features a pan-tilt unit (PTU; PTU-D46-17.5 W, Directed Perception, Burlingame, CA, USA), which allows the sensor head to record data at any view zenith angle (*θ_v_*) between 43° and 68° and at a view azimuth angle (*φ_v_*) between ± 170° from the initial position for scaling of the observations through modeling of the bidirectional reflectance distribution function (BRDF; [27]) and investigation of foliage clumping effects [33]. In addition, the ability to programmatically define *θ_v_* and *φ_v_* permits users to automatically compare tower-based measurements to satellite observations without further modeling of sensor geometries difference, with footprint size on the ground depending on *θ_v_* and height of installation.

**Table 1:** Technical specifications of the third generation Automated Multiangular SPectro-radiometer for Estimation of Canopy reflectance system (AMSPEC-III).

Feature	AMSPEC I [7]	AMSPEC II [8]	AMSPEC-III
Spectro-radiometer	Unispec-DC		JAZ-COMBO
Spectrum (nm)	350–1200		200–1100
Resolution (nm)	3.3		0.145
Repeatability (nm)	0.1		0.23 at 730 nm
Integration time (s)	0.004–3.28		0.001–65 (20 typical maximum)
Averaging number of scans	1000 at 0.4 s (less for longer ITs)		100 scan/s
Operation temperature (°C)	0–40		0–55
Scan time (s)	2–6		2–6

Communication between computer and spectro-radiometers and between computer and PTU is ensured via serial connections and USB standard for UDC and JC, respectively; the webcam is connected via local area network (LAN). The system can be linked to an external network or mobile communication device to allow remote access to the data.

Table 2 contains the AMSPEC-III components and their approximate costs at the time of the design of the research. The costs have not significantly changed at the time of writing this manuscript. The total sensor cost is the result of the individual components and labor costs, not included in Table 2. The majority of the costs is associated with the spectro-radiometers, with JC resulting in approximately a third of the UDC cost although requiring a temperature-controlled housing due to higher sensitivity to differences in temperature.

**Table 2:** AMSPEC-III components and approximate costs.

Item	Provider	Qty	Cost (USD)
Unispec-DC	PP-Systems, 110 Haverhill Rd, Suite 301, Amesbury, MA 01913, USA	1	22,750
JAZ-COMBO	Ocean Optics, 830 Douglas Ave, Dunedin, FL 34698, USA	1	6860
NetCam SC, 5 MP	Stardot Tech., 6820 Orangethorpe Ave, Buena Park, CA 90620, USA	1	1340
PTU-D46-17	Directed Perception, 890 C Cowan Rd, Burlingame, CA 94010, USA	1	2340
Computer (ARK-1122H-S6A1E)	Advantech, 380 Fairview Way, Milpitas, CA 95035, USA	1	600
External hard drive (840 PRO SSD, 128 GB)	Samsung Electronics Co., Ltd., 95, Samsung 2-ro, Giheung-gu, Yongin-si, Gyeonggi-do, Korea, 446-811	1	150
Box	–	1	500
Mounts, misc	–	-	750

### 2.2. Field Site Description

**Figure 1:**
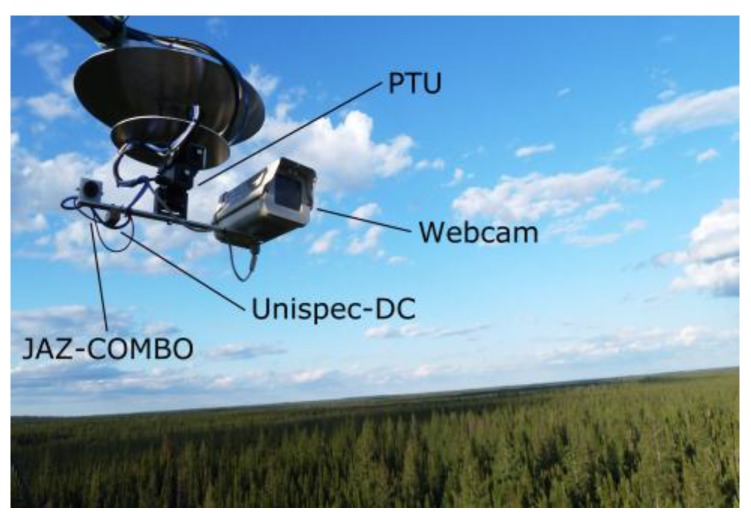
*In situ* photograph of the third generation Automated Multiangular SPectro-radiometer for Estimation of Canopy reflectance system (AMSPEC-III) taken at the Southern Old Black Spruce (SOBS) site.

The dual spectro-radiometer AMSPEC-III system was installed in April 2013 at the Southern Old Black Spruce (SOBS) site; CO_2_ measurements were taken with EC as part of the Boreal Ecosystem Research and Monitoring Sites (BERMS) initiative [34]. The site is located ~100 km NE of Prince Albert, Saskatchewan (Canada) at 598 m above sea level (latitude 53.9872° N, longitude 105.1178° W) at the southern edge of the boreal forest, and it is dominated by black spruce (*Picea mariana* (Mill.)) up to 11 m high (average height 7.2 m; [35]), with occasional tamarack, 10–16 m high, and jack pine, 13 m high. The canopy is approximately 135 years old and with leaf area index 5.6 [35]. SOBS was selected among other BERMS sites because showing the highest photosynthetic capacity under optimal conditions [36]. The tower module was mounted on a scaffold tower at 25 m height, approximately 18 m above the canopy. The final setup of the tower module at SOBS is shown in Figure 1.

### 2.3. Data Processing

**Figure 2:**
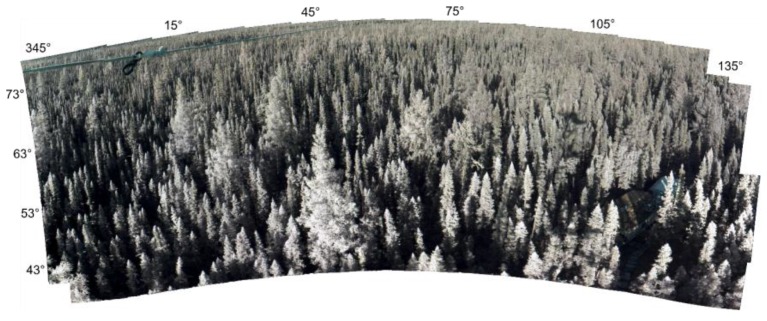
Image composite at SOBS over a ~165° observation cycle. The photographs have been stitched from 57 individual observations (340° ≤ *φ_v_* ≤ 145°) using Microsoft^®^ Research Image Composite Editor.

The AMSPEC-III system records solar irradiance and canopy radiance simultaneously to the sensors viewing geometry, solar position, time of measurement and the webcam RGB image. The PTU movement was set to 10° horizontal steps, completing a full rotation every 30 min. At each horizontal location, four different vertical angles were measured, alternating between *θ_v_* ∈ {48°; 58°; 68°; 78°} and *θ_v_* ∈ {43°; 53°; 63°; 73°} every 15 min (half full rotation). A measurement in the solar plane was performed at the beginning of every 15 min cycle. A portion is not seen by the instrument due to obstruction from the scaffold tower, and depends on the tower size and how the instrument is installed on it. At SOBS, we excluded the measurements with φ_v_ comprised between 145° and 180°, and between 220° and 340° in order to exclude obstructed images. An example of a ~165° observation cycle at SOBS is in Figure 2.

By design, the use of normalized difference indices should compensate some of the differences in light sensitivity between the upward- and downward-looking channels (*i.e*. irradiance and radiance, respectively). However, light sensitivity depends also on data acquisition wavelength. For this reason, a calibration before and during data acquisition was performed using a Labsphere^®^ diffuse reflectance target [37]. After considering the sensor’s differences in sensitivity to light due to the individual photodiodes and fiber optics used in the system, for dual-channel radiometers the measured canopy reflectance (ρ) is defined as the ratio of canopy radiance and solar irradiance. Differences in light sensitivity can be corrected through a cross-calibration approach by measuring the reflectance of the standardized reference target [6]:

$$\rho =\frac{L\cdot I\prime }{I\cdot L\prime }$$

where L is the measured radiance of the canopy sensor, I is the simultaneously measured irradiance, L′ is the measured radiance of the control surface, and I′ is the irradiance at the time L′ was measured.

Neither UDC nor JC provides an internal shutter mechanism to automatically correct for dark current (dc), defined as the electrical current that generated by thermal electrons in the photocathode of optical instruments [38]. For this reason, the acquired data can only be corrected in a post-processing step using manual measurements taken with both sensors completely covered from light. Using previous AMSPEC versions, Hilker *et al*. [7,8] demonstrated a relationship between the sensor’s temperature (as measured by the internal thermometer of the spectro-radiometer) and the dc measured when blocking off the light from both sensors, thereby allowing an automated correction of this drift in the measured sensor radiance.

For this study, we analyze simultaneous UDC and JC measurements at SOBS. In order to directly compare the two systems, the JC channels were spectrally resampled to UDC using the arithmetic mean of overlapping wavebands. We present radiance, irradiance and calculated reflectance over one sample day (1 September 2013) in three spectral channels (*i.e*., 559 nm; 660.3 nm; 809.7 nm) representative of green, red and NIR light, respectively. To avoid possible sky contaminations, all measurements with *θ_v_* > 63° were excluded from the dataset. We focus the results on the retrieval of the Photochemical Reflectance Index (PRI; [23]), as well as demonstrate the directional effects using three spectral channels: green (559 nm), red (660.3 nm) and NIR (809.7 nm).

## 3. Results

**Figure 3:**
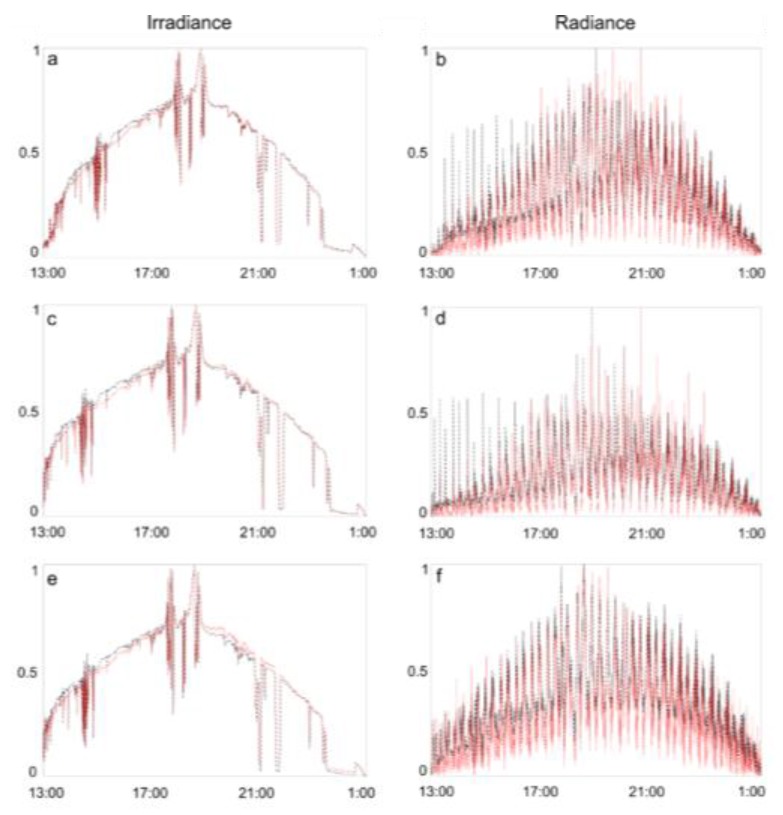
Green (**a**,**b**), red (**c**,**d**) and NIR (**e**,**f**) normalized irradiance and radiance measured with UDC (dashed black) and JC (dotted red). Since both instruments provide raw measurements in arbitrary units, the data shown here were linearly rescaled between minimum and maximum values (0–1). Time is GMT (hh:mm).

**Figure 4:**
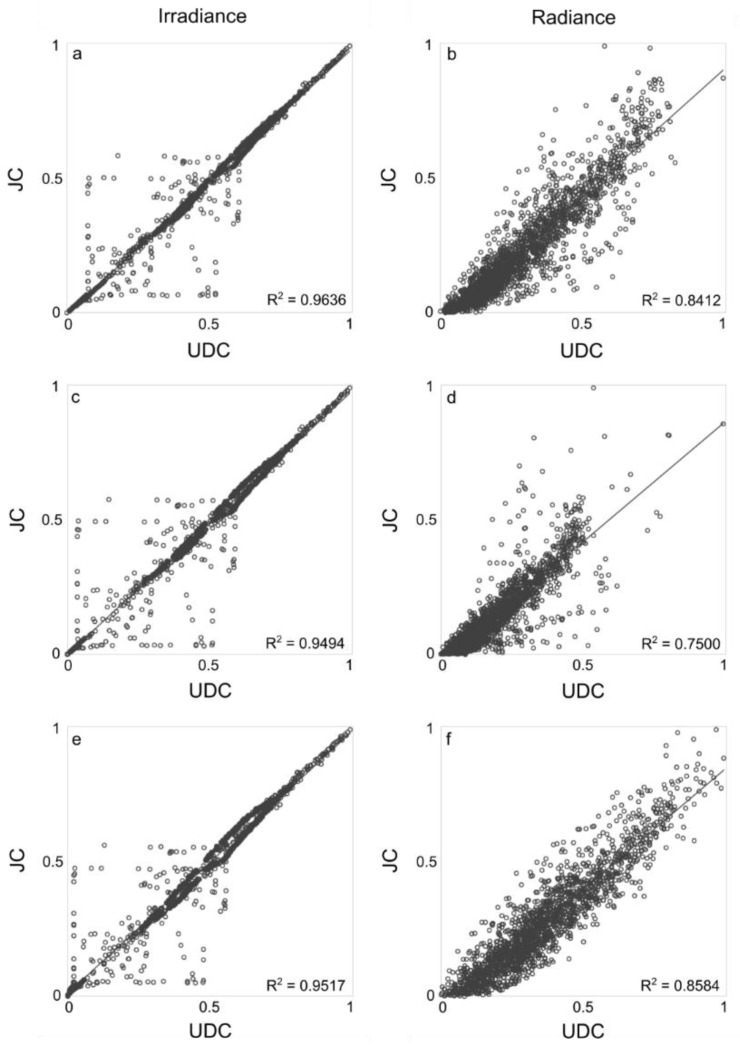
Scatterplots of green (**a**,**b**), red (**c**,**d**) and NIR (**e**,**f**) irradiance and radiance measured with UDC and JC. The linear regression lines are in solid.

A comparison of the measured green, red and NIR radiance and irradiance is in Figure 3, with respective scatterplots are in Figure 4.

**Figure 5:**
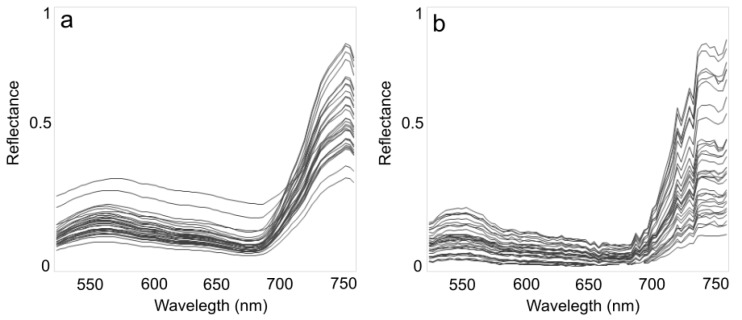
Spectra for the observation cycle in Figure 2 (340° ≤ *φ_v_* ≤ 145°) from (**a**) UDC and (**b**) JC.

Figure 5 shows an example of UDC and JC spectra acquired on 1 September, 2013 over the observation cycle in Figure 2. The variability in reflectance is largely due to the sun-observer geometry (*cf.* [7,8]) as the sensor observes the same location around the flux tower every 30 min.

**Figure 6:**
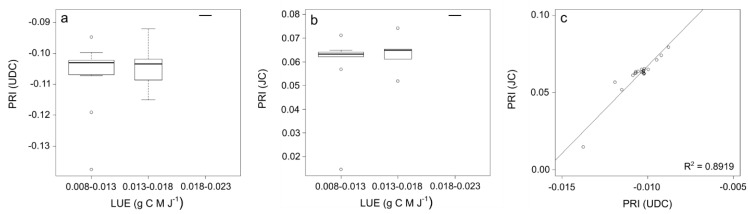
Box-and-whisker plot of 30 minutes EC-measured LUE and PRI observed by (**a**) UDC and (**b**) JC, with box width proportional to the number of observations. (**c**) UDC and JC measured PRI linear regression with regression line in solid.

One of the main applications of previous AMSPEC installations was the determination of photosynthetic light use efficiency (LUE), which describes how efficiently a plant converts the absorbed solar radiation into biomass [39,40]. Figure 6 shows the comparison between half hourly EC-measured LUE and the PRI observed by UDC and JC at SOBS on 1 September, 2013.

**Figure 7:**
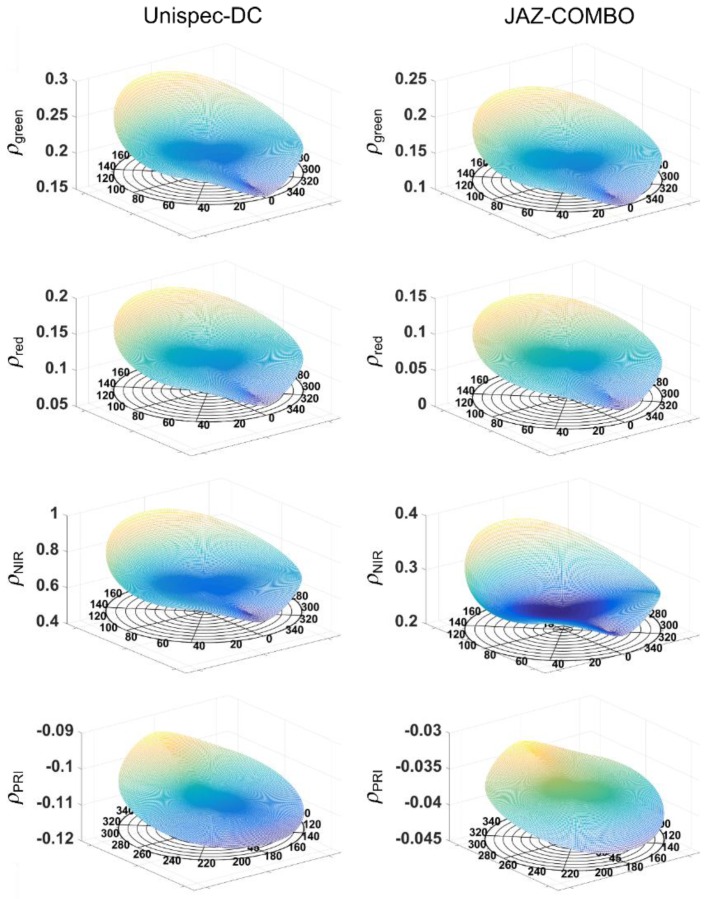
BRDF models for UDC and JC.

Figure 7 illustrates the bi-directional reflectance distribution measured using off-nadir observations from both spectrometers, using a semi-empirical kernel approach [41,42]. Kernel based BRDF models are one of the most commonly used methods to describe BRDF effects. These functions represent angular reflectance distribution as linear combination of basic BRDF shapes describing volumetric and geometric scattering effects [42]. Their simple character allows acquisition of model parameters from mathematical inversion of relatively few reflectance observations, thereby facilitating applications over a wide range of spatial scales. Examples of BRDF models for UDC and JC recorded on 1 September, 2013 at SOBS are shown in Figure 7.

## 4. Discussion and Conclusions

Upscaling of spatially discrete observations to landscape and global scales is crucial to developing better insights into the carbon cycle. However, due to the different nature of tower-based EC and satellite systems, this task remains challenging. The high spatial, spectral and temporal resolution of the tower-based AMSPEC systems (*cf.* [7,8]) allows to observe vegetation canopy under different view and sun angles for a comprehensive analysis of spectral reflectance at the stand level (e.g., [43,44]), of fundamental importance for the interpretation of satellite observations.

Initial findings show a good correspondence between UDC and JC irradiance and radiance, with R^2^ comprised between 0.75 and 0.85 (Figure 3 and Figure 4), with JC reflectance spectra noisier than UDC (Figure 5) due to the different spectral resolution (*cf.* Table 1; [45]). This suggests that JC can successfully be used to substitute more expensive measurement units such as the UDC, although extreme regions of the spectral range of each instrument may not be reliable (e.g., Figure 5). Figure 6 does not show a strong regression fit between half hourly EC-measured LUE and PRI measured by neither UDC and JC; however, the linear regression between PRI measured with the two spectro-radiometers shows a very good correspondence (R^2^ = 0.89). By design, AMSPEC-III allows to characterize the BRDF, an essential requirement to scale measurements across different view and sun angles (e.g., Figure 2) when detecting physiologically induced changes in spectra. The similarity of the BRDF models (Figure 7) highlight the potential of JC to derive year-round estimates multi-directional PRI measurements from AMSPEC III instrumentation (e.g., Figure 7). In addition, the webcam implemented in the AMSPEC-III system will help to further analyze the impact of phenological changes on vegetation canopy spectral reflectance and the correspondent stand-level photosynthesis.

Based the results presented in this work, we believe that AMSPEC-III equipped with JC is a powerful tool for investigating these stand-level relationships and detecting photosynthesis and canopy spectra. Despite JC is more sensitive to differences in temperature and requires a temperature-controlled housing and therefore increases also the energy requirements for the system, the majority of the AMSPEC-III system costs is associated with the UDC spectro-radiometer (Table 2). However, the performance obtained by JC and presented in this study justify the choice of JC over UDC for network of sensors for monitoring of vegetation physiology.

Interoperability of different tower based instrumentation is an important prerequisite of tower based spectral networks [21], especially when composed of instrumentation provided by multiple investigators and funding sources. Our study has demonstrated the interoperability of the UDC spectro-radiometer and the more cost effective JC used in an AMSPEC-III system. On the other hand, simpler low cost instruments are available as well. For example, QuadPod is capable to quantify NDVI and PRI [46] at a fraction of the cost of AMSPEC. However, these highly specialized sensors are dedicated to the measurement of specific indices and cannot be implemented in a multi-angular setup. In addition, the collection of spectra over multiple bands will allow to analyze various vegetation indices.

Cost effective solutions, such as the JC instrumentation used in this study, could make important contributions to tower based remote sensing networks and therefore to scaling forest ecosystem productivity from stand to satellite and global scales. For instance, combined effort of several AMSPEC-III systems acquiring spectra from multiple flux tower sites simultaneously, including spectral observations of various vegetation types in different ecosystems, will allow to considerably improve our understanding of the interactions between vegetation physiology and spectral characteristics, helping to calibrate broad-scale observations to stand-level measurements, and ultimately lead to improved understanding of changing vegetation spectral features from satellite.
